# Repurposing the anti-malarial drug, quinacrine: new anti-colitis properties

**DOI:** 10.18632/oncotarget.10608

**Published:** 2016-07-14

**Authors:** Alexander A. Chumanevich, Erin E. Witalison, Anusha Chaparala, Anastasiya Chumanevich, Prakash Nagarkatti, Mitzi Nagarkatti, Lorne J. Hofseth

**Affiliations:** ^1^ Department of Drug Discovery and Biomedical Sciences, South Carolina College of Pharmacy, University of South Carolina, Columbia, SC, USA; ^2^ School of Medicine, University of South Carolina, Columbia, SC, USA

**Keywords:** quinacrine, colitis, inflammation, dextran sulfate sodium, oxazolone

## Abstract

**Background:**

Ulcerative colitis (UC) is a chronic inflammatory bowel disease that is associated with an increased risk of colorectal cancer in 8-10 years after disease onset. Current colitis treatment strategies do not offer a cure for the disease, but only treat the symptoms with limited success and dangerous side-effects. Also, there is no preventive treatment for either UC or colorectal cancer. Quinacrine is an anti-malarial drug with versatile use in the treatment of diseases involving inflammatory response such as rheumatoid arthritis and lupus erythematosus. It also has putative anti-cancer effect. Quinacrine's anti-inflammatory, anti-oxidant properties, and anti-tumorigenic properties make it a potential small molecule preventive agent for both UC and associated colorectal cancer.

**Results:**

There were obvious changes in the CDI, histology, and inflammatory load in quinacrine-treated groups in a dose and time dependent manner in both models of UC, induced by chemical or haptenating agent.

**Methods:**

We tested quinacrine at two different doses as a colitis treatment agent in two mouse models of UC - the dextran sulfate sodium and oxazolone. The clinical disease index (CDI), histological changes of the colon, levels of inflammatory markers (Cox-2, iNOS, p53) and overall health vitals were evaluated.

**Conclusions:**

We demonstrate that quinacrine successfully suppresses colitis without any indication of toxicity or side-effects in two mouse models of UC.

## INTRODUCTION

Ulcerative colitis (UC) is a chronic disease that causes inflammation and ulcers in the colon and the rectum. Colorectal cancer is the most serious complication of ulcerative colitis [1]. The risk of developing colorectal cancer increases after 8-10 years of colitis at a rate of 0.5 - 1% for every year of disease duration [2]. The severity of the disease also has a significant impact on the transformation of the disease into cancer [3]. Current medications only help in alleviating the symptoms, but for the most part results are modest and there are dangerous side effects.

Quinacrine (IUPAC name 4-N-(6-chloro-2-methoxyacridin-9-yl)-1-N,1-N-diethylpentane-1,4-diamine), approved by the FDA, is a heterocyclic three-ring compound that was widely used during World War II as an anti-malarial agent. Over the last half century, it has been used for the treatment of giardiasis, tapeworm infestations, and connective tissue diseases, such as lupus erythematosus and rheumatoid arthritis [4-8]. Mechanistically, it is becoming increasingly apparent that quinacrine targets several key players involved in inflammation and the inflammation-to-cancer sequence [9]. For example, quinacrine activates p53 and superoxide dismutase, and inhibits NF-κB and phospholipase A2 [10-15]. Anti-cancer properties include the induction of apoptosis and cell cycle arrest in cancer cells [14-17], as well as the inhibition of Wnt-TCF signaling [18] and topoisomerase activity [19]. Quinacrine can also modify the expression of microRNAs involved in carcinogenesis [13].

Recognizing these anti-cancer and anti-inflammatory properties of quinacrine, we hypothesized that quinacrine can suppress colitis in mice. In this article we test this hypothesis in two different mouse models: a DSS model, and Oxazolone model of colitis, showing a significant decrease in the overall colonic inflammatory load in the quinacrine treated animals.

## RESULTS

### Quinacrine downregulates iNOS in murine macrophages cell line

**Figure 1:**
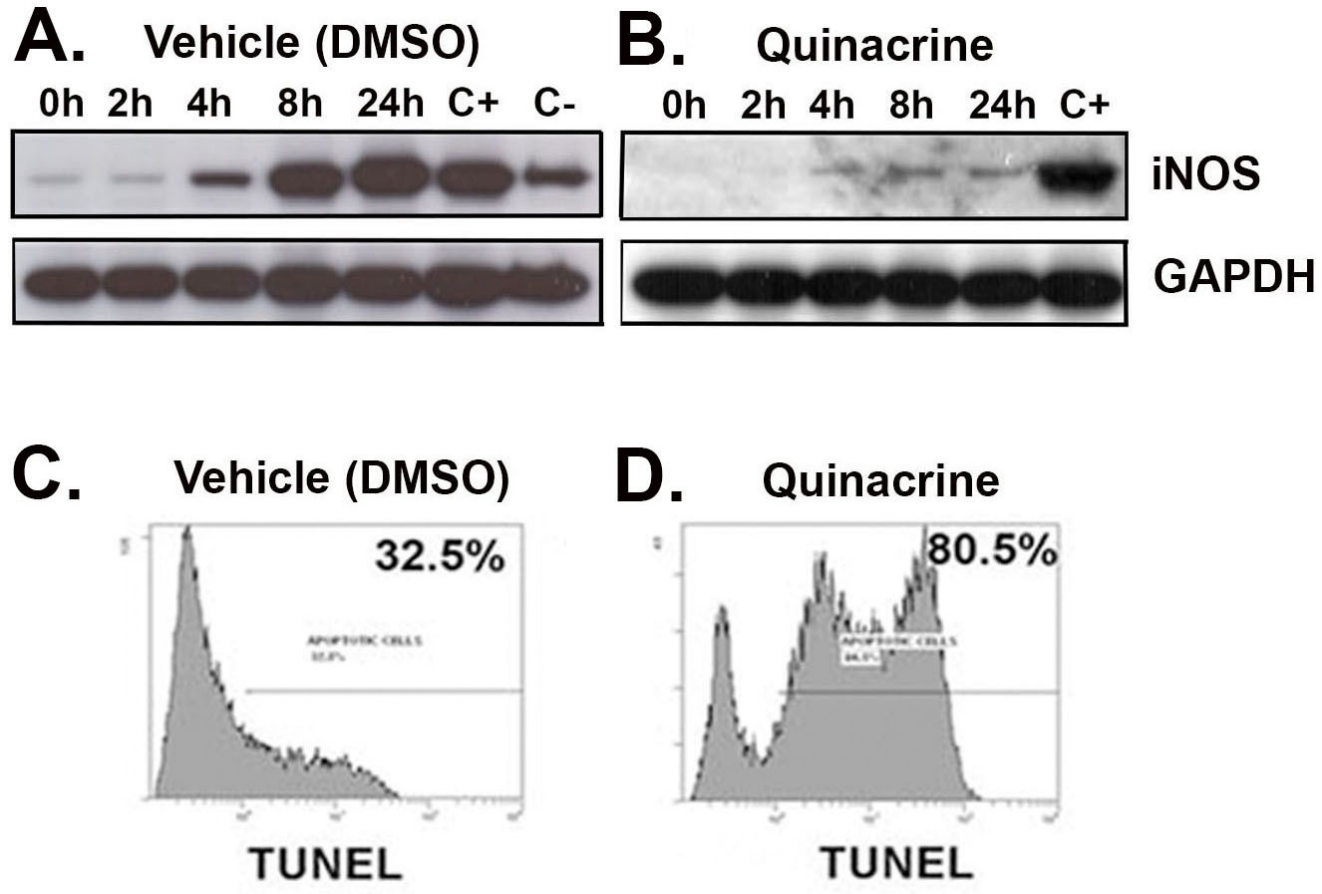
Quinacrine suppresses the activation of iNOS and induces apoptosis *in vitro* ANA-1 mouse macrophages were pre-incubated for 12 h with 100 μg/ml of quinacrine **B.** or a vehicle (DMSO) **A.** Quinacrine was washed off, and then media containing 100 μg/ml IFN-γ to stimulate cells were added. Following the indicated incubation time, cells were harvested for protein and examined for a response with iNOS being the inflammation marker. The positive control (C+) is *ANA-1* cells not exposed to quinacrine (on panel B) or vehicle (on panel A) at 24h time point. The negative control (C-) corresponds to the ANA-1 cells exposed to vehicle only at 24h time point. GAPDH was probed for the background and loading control. For apoptosis **C.** **D.**, TK6 lymphoblastoid cells were exposed to vehicle (DMSO) (C), or 100 ug/ml quinacrine (D) for 12 h, then assessed for apoptosis by TUNEL labeling.

The cellular levels of nitric oxide (NO) and associated reactive nitrogen species (RNS) are driving factors of inflammation and cancers associated with chronic inflammation [20]. Since quinacrine has putative anti-inflammatory properties, we tested its ability to affect NO synthesis *in vitro*, using the ANA-1 murine macrophages cell line*.* Western blot analysis shows that pre-incubation of ANA-1 cells with quinacrine effectively attenuates the induction of iNOS for up to 24 hours (Figure 1A and 1B).

### Quinacrine stimulates inflammatory cell apoptosis

We have previously shown that compounds that both: (a) suppress iNOS induction in inflammatory cells; and (b) induce apoptosis in inflammatory cells *in vitro*; are highly likely to suppress colitis *in vivo*. We have demonstrated this with Resveratrol, American Ginseng, a protein arginine deiminase inhibitor, Chlor-Amidine, and with a Hexane Fraction of American Ginseng [21-26]. Therefore, before testing the efficacy of quinacrine against colitis *in vivo*, we first asked whether quinacrine can induce apoptosis in inflammatory cells. Consistent with this understanding, Figure 1C and 1D show quinacrine induces apoptosis in ANA-1 mouse macrophages.

### Quinacrine suppresses colitis in DSS mouse model in both dose- and time- dependent manner

UC is associated with long lasting inflammation in the bowels. Based on our *in vitro* studies where quinacrine suppresses iNOS induction and drives apoptosis of inflammatory cells, we have tested it on mice to check if it suppressed DSS induced colitis. Quinacrine was given to mice *ad libitum* at the two doses of 50 mg/kg and 10 mg/kg equivalent in the water after 1 week exposure to 2% DSS. The mice that received no quinacrine treatment showed moderate to severe inflammation and mild ulceration with a histology score rising gradually over the exposure time from 7.8 ± 1.04 at 7 days to 26.2 ± 1.7 at 14 days and to 27.11 ± 1.6 at 17 days time points. In contrast, although colitis did not return to that of the water-only treated group, the quinacrine-treated mice demonstrate mild inflammation and ulceration at both doses, as reflected by histology score of 16.5 ± 3.0 for the quinacrine dose of 10 mg/kg and 13.1 ± 2.6 for the quinacrine dose of 50 mg/kg at the 14 day time point (Figure 3). Interestingly, further treatment with quinacrine at a dose of 50 mg/kg reduces inflammation score even more - to 9.9 ± 2.2 at 17 days time point. Multiple additional end points support this conclusion (Table 1). Since mouse colon length decreases in an inflamed state, we also used this parameter as a measure for inflammation severity. For the DSS-treated group, the average colon length was 7.0 ± 0.2 cm at day 14, and even shorter for the 17 day time point - 6.5 ± 0.2 cm. In contrast, the average colon length of the DSS + Quinacrine group was 8.4 ± 0.3 cm for 10 mg/kg and 8.6 ± 0.3 cm for 50 mg/kg dosages at 14 days, which is reaching the mean colon lengths in the water-alone group (9.7 ± 0.3) at the same time point. The average clinical disease index (CDI), which reflects overall health status of the animals based on observed weight loss, diarrhea and hemoccult (blood presence in the stool), clearly shows therapeutic effect of quinacrine on colitis progression - average CDI of animals, treated with 50mg/kg dose of quinacrine for 10 days after colitis onset, reduced more than twice, from 8.9 ± 0.1 to 4.0 ± 0.8. Similarly spleen volumes and white blood cell counts (reflecting systemic inflammation), and body weight recovered with quinacrine treatment (Table 1). Overall, these results are consistent with the notion that quinacrine suppresses DSS-induced colitis in time- and dose- dependent manner.

**Table 1:** Gross characteristics of treated groups in DSS model

Treatment	Weight change, g	Colon Length, cm	CDI	Spleen, mm^3^	WBC Count, m/mm^3^†	RBC Count, b/mm^3^††
Water	1.6 ± 0.0	9.7 ± 0.3	0.2 ± 0.1	101.5 ± 17.8	7.4 ± 0.4	9.5 ± 0.2
2% DSS (7 days)	-0.4 ± 0.5	8.0 ± 0.2	5.5 ± 0.9*	199.8 ± 19.3	8.9 ± 0.5	10.0 ± 0.2
2% DSS (14 days)	-2.1 ± 0.5	7.0 ± 0.2	6.3 ± 0.1	274.3 ± 35.5	10.6 ± 0.5	8.8 ± 0.2
2% DSS (17 days)	-2.6 ± 0.69	6.5 ± 0.21	8.9 ± 0.1	275.4 ± 14.6	8.6 ± 0.5	8.9 ± 0.2.
2% DSS + 50 mg/kg of Quinacrine (14 days)	-0.6 ± 0.6	8.6 ± 0.3	4.2 ± 0.3*	152.4 ± 13.2	9.5 ± 0.6	9.18 ± 0.2
2% DSS + 50 mg/kg of Quinacrine (17 days)	-1.6 ± 0.4	8.2 ± 0.3	4.0 ± 0.8*	162.7 ± 27.1	6.1 ± 0.4	9.3 ± 0.4
2% DSS + 10 mg/kg of Quinacrine (14 days)	-1.5 ± 0.5	8.4 ± 0.3	4.9 ± 0.8*	163.9 ± 9.7	9.6 ± 0.4	8.57 ± 0.2

Values are group averages ± SE. All values are significantly different from corresponding 2% DSS only and Water groups.

WBC, white blood cells. RBC, Red Blood Cells. † Millions per cubic milliliter of blood. †† Billions per cubic milliliter of blood.

### Quinacrine suppresses colitis in oxazolone mouse model

Although the DSS model of colitis is extremely useful in studying the therapeutic effects of small molecules against inflammation in the colon, a pitfall is that it is a chemically-induced model. Therefore, we have tested quinacrine in an alternative model - oxazolone model of colitis. We have followed Wirtz *et al.* protocol [27] with some modifications. While the first part of the animals were sacrificed in 5 days after oxazolone administrations (Oxazolone 5/5 groups), an additional dose of oxazolone was administered on day 5 to the second cohort of animals, which were also sacrificed in 5 days after the last oxazolone administration (groups Oxazolone 5/10). Similarly to DSS model, quinacrine was also given to mice *ad libitum* at the two doses of 50 mg/kg and 10 mg/kg equivalent in the water 8 h after an intra-rectal exposure to oxazolone and continued to the sacrifice time point. Mice that did not receive any quinacrine treatment developed moderate colitis by experimental day 10 with average inflammation score of 20.8 ± 3.4 (group Oxazolone 5/5), which progressed to a more severe state by experimental day 15 as indicated by average inflammation score of 28.5 ± 5.6 (group Oxazolone 5/10) (Figure 4A). In contrast, quinacrine drastically reduces severity of colitis in treated mice, resulting in reduction of inflammation scores to 5.4 ± 1.6 and 2.7 ± 0.7 for 50 mg/kg dose in Oxazolone 5/5 and Oxazolone 5/10 groups, correspondingly. The lower 10 mg/kg dose of quinacrine was also very effective for short term experimental group Oxazolone 5/5, reducing inflammation scores to 5.3 ± 1.7, and less, but still effective, in the long term run - 8.1 ± 1.5 for 10 mg/kg Oxazolone 5/10 groups. The substantial suppression of colitis in quinacrine treated mice was also reflected in all major parameters including colon length, weight change during the experiment, spleen volume and CDI (summarized in Table 2). For example, the CDI for quinacrine treated mice was almost 7 times lower for the dose of 50 mg/kg, and 4 times lower for the dose of 10 mg/kg for the Oxazolone 5/10 groups.

**Table 2:** Gross characteristics of treated groups in Oxazolone model

Treatment	Weight change, g	Colon Length, cm	CDI	Spleen, mm^3^
Water (5 days)	0.5 ± 0.1	8.9 ± 0.8	0.2 ± 0.2	141.5 ± 16.1
Water (10 days)	1.6± 0.3	9.7 ± 0.3	0.3 ± 0.1	101.5 ± 6.3
Oxozolon 5/5	-1.7 ± 1.44	7.5 ± 0.2	8.1 ± 0.6	253.2 ± 19.3
Oxozolon 5/10	-2.1 ± 1.0	7.7 ± 0.8	6.7 ± 1.3	192.1 ± 20.7
Oxazolon 5/5 + 10 mg/kg of Quinacrine	1.41 ± 0.3	8.3 ± 0.3	1.71 ± 0.8	139.8 ± 10.9
Oxazolon 5/5 + 50 mg/kg of Quinacrine	-0.5 ± 0.8	9.4 ± 0.4	2.9 ± 1.0	146.7 ± 13.2
Oxazolon 5/10 + 10 mg/kg of Quinacrine	0.1 ± 0.4	8.5 ± 0.3	2.0 ± 0.7	175.4 ± 25.8
Oxazolon 5/10 + 50 mg/kg of Quinacrine	-0.5 ± 0.2	9.9 ± 0.3	1.0 ± 0.6	105.9 ± 10.2

Values are group averages ± SE. Values are significantly different from corresponding Oxazolone only and Water groups.

Overall, treatment of mice with quinacrine in oxazolone model of colitis leads to suppression of colitis in both time-and dose dependent manner, similarly to DSS model.

### Markers of inflammation and inflammatory stress are reduced in quinacrine-treated mice

**Figure 2:**
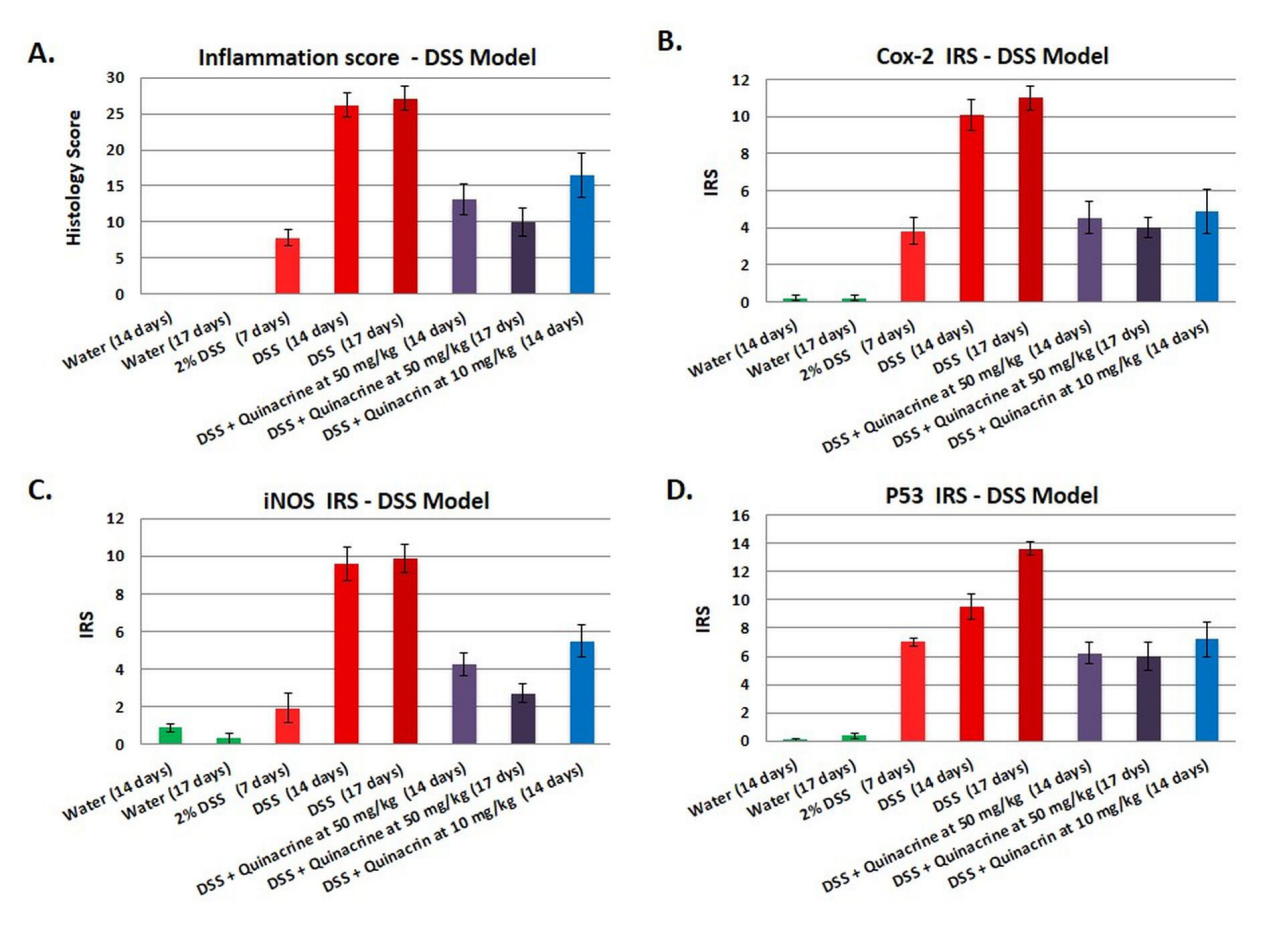
Effects of the quinacrine on the colon histology score, Cox-2 immunoreactivity score, iNOS immunoreactivity score, and p53 immunoreactivity score in the DSS model of colitis Values represent the average scores for each group (*N* = 10-17 per group). All values are statistically significantly different (*p* < 0.05) from the other groups.

To further assess the impact of quinacrine on inflammatory markers *in vivo*, we examined Cox-2, iNOS, and p53 expression in the colon of mice. Immunohistochemical staining was accomplished by rocking slides using the Antibody Amplifier^TM^ (ProHisto, LLC) to ensure even, consistent, sensitive and reproducible staining. Figure 2B-2D show quantification of each endpoint for DSS model, and 3B-D - for oxazolone model. Overall, Cox-2 (Figures 2B, 4B), iNOS (Figures 2C, 4C), and p53 (Figures 2D, 4D) levels were elevated in both models' mice, with most staining appearing in the inflammatory cells. Cox-2 and iNOS staining were statistically significantly reduced in the quinacrine-treated mice; there was also a trend to decreasing p53 levels. Figure 5 shows representative sections of each endpoint as indicated. Such results reflect a reduction in the number of inflammatory cells (that otherwise are expressing these inflammatory markers), and complement our H&E histopathology results.

**Figure 3:**
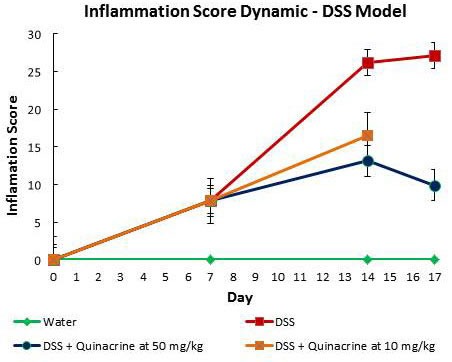
Dynamic of inflammatory score in DSS model of colitis

**Figure 4:**
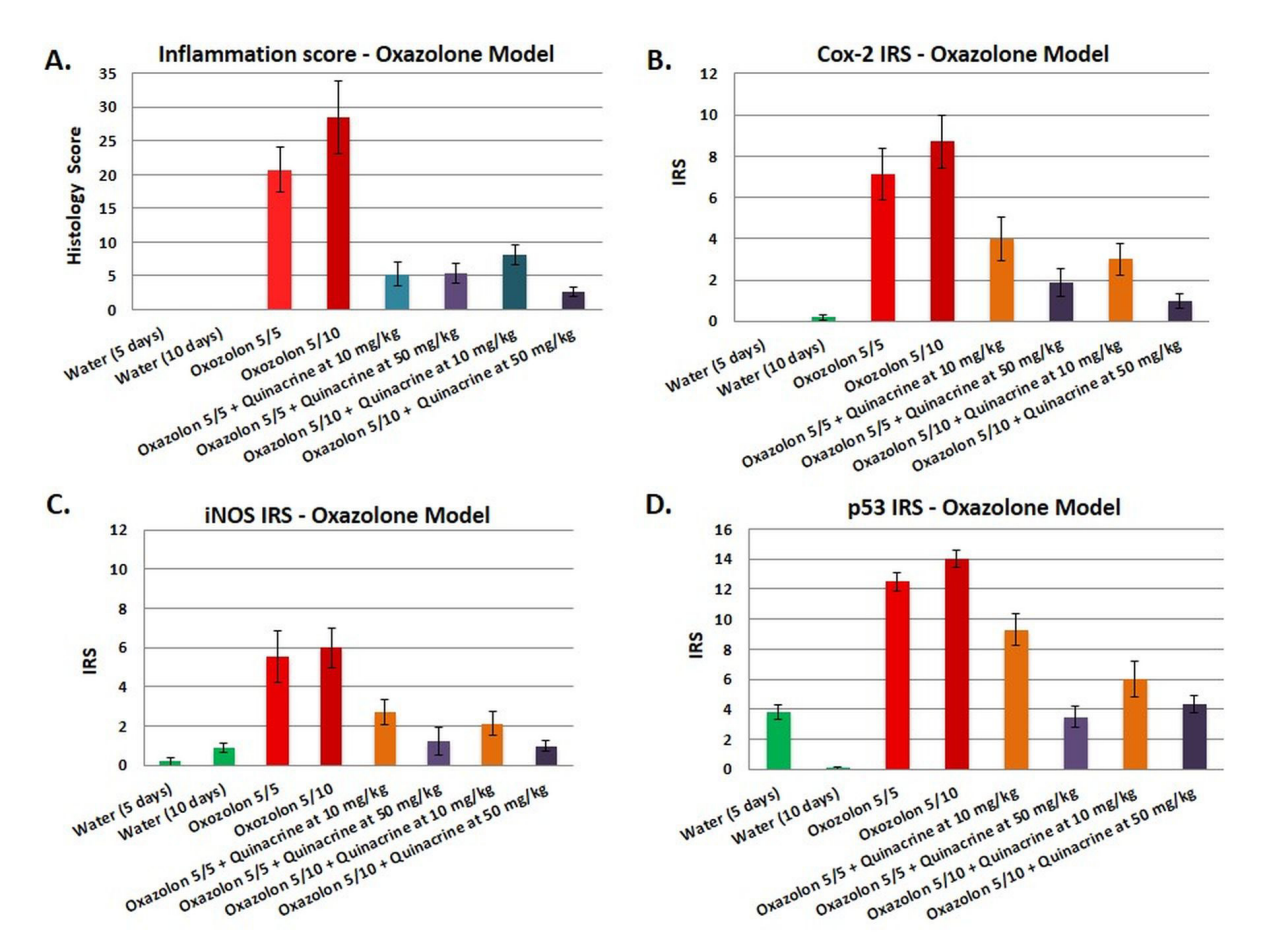
Effects of the quinacrine on the colon histology score, Cox-2 immunoreactivity score, iNOS immunoreactivity score, and p53 immunoreactivity score in oxazolone model of colitis Values represent the average scores for each group (*N* = 6-8 per group). All values are statistically significantly different (*p* < 0.05) from the other groups.

**Figure 5:**
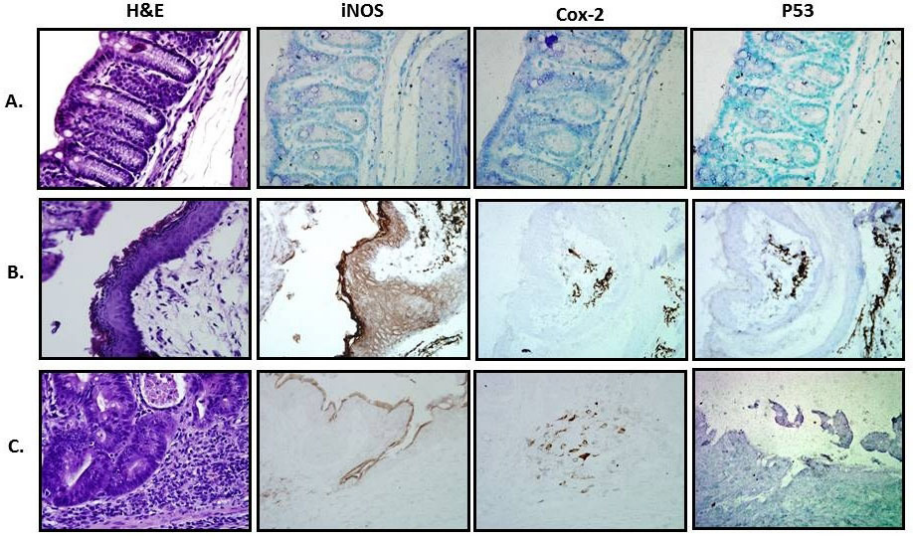
Representative histological and IHC sections from treated groups **A.** Water; **B.** 2% DSS; **C.** 2% DSS + 50 mg/kg Quinacrine. 400x magnification.

## DISCUSSION

UC is a painful disease and the complications of increased risk of colorectal cancer only add to the distress. Other than surgery, treatment available now can only alleviate the symptoms, giving temporary relief, but cannot cure the disease. Our search for a novel treatment for this disease led us to quinacrine; widely used as anti-malarial and anti-protozoal drug. Since its discovery for use as an anti-parasitic, multiple studies have identified a putative anti-inflammatory mechanism for quinacrine [12, 13, 28]. With colon inflammation as a hallmark of UC, we hypothesized that quinacrine can suppress colitis in a mouse model of UC. Results we report here are consistent with this hypothesis.

We present that quinacrine suppresses the induction of iNOS *in vitro* (Figure 1), and iNOS, Cox-2 and p53 *in vivo* in two independent mouse models of colitis (Figures 2 and 3). Others have shown that quinacrine disrupts additional mediators of inflammation, including phospholipase A2, formation of prostaglandins, and the arachidonic acid cascade [29, 30]. Also, quinacrine inhibits NF-κB, TNFα, and IL-1β [14, 31]. Such results give insight into the potential mechanisms of quinacrine in suppressing UC. Importantly, anti-TNF therapy is a mainstream treatment for patients with moderate to severe inflammatory bowel disease [32]. There is also molecular evidence that iNOS, Cox-2 and p53 play a role in experimental colitis and human colitis [23, 33-37]. Furthermore, IL-1β has been identified as a target for Inflammatory Bowel Disease treatment in multiple studies [38-40]. Mechanistically, IL-1β promotes innate immune pathology in intestinal inflammation by augmenting the recruitment of granulocytes and the accumulation and activation of innate lymphoid cells [41]. Therefore, suppressing IL-1β signaling has anti-inflammatory consequences. Finally, NF-kB signaling in myeloid cell appears to be critical for the development of colitis and colon cancer associated with colitis [42, 43].

Failure to regulate inflammatory responses in the intestinal or colonic mucosa leads to an inappropriate, sustained, and injurious immunologic reaction. A key mechanism for immune suppression is apoptosis of overly aggressive inflammatory cells and defects in inflammatory cell apoptosis are likely to be critical in the pathogenesis of colitis [44, 45]. We present evidence that quinacrine also induces apoptosis in inflammatory cells. In this context, others have also shown a pro-apoptotic impact of quinacrine on other cell types, including colon cells, as an anti-cancer mechanism [14, 17]. Such studies then identify an additional potential mechanism of quinacrine in the suppression of colon inflammation and the possible prevention of colon cancer.

Overall, quinacrine has a rich history as an anti-protozoal [46], anti-malarial [47], anti-rheumatic [48], anti-prion [49], anti-cancer [50], and an intrapleural sclerosing agent [50]. Consequently, it has potential or proven use against malaria [47], giardiasis [6], pneumothorax [51], Creutzfeldt-Jakob disease [52], cancer [5, 53], and in particular, female sterilization [50, 54]. Importantly, it also shows possible efficacy against rheumatoid arthritis [55] and lupus [56]. Because of this efficacy against arthritis and lupus (autoimmune diseases), and the ability of quinacrine to induce inflammatory cell apoptosis, it is not that surprising that quinacrine shows efficacy against another autoimmune disease: colitis. Future studies will determine efficacy in other models of colitis, and may eventually show efficacy against inflammatory bowel diseases in humans.

## MATERIALS AND METHODS

### Chemicals and reagents

Quinacrine dihydrochloride and oxazolone were obtained from Sigma. Dextran sulfate sodium (molecular weight, 36,000-50,000) was purchased from MP Biomedicals.

### Cell lines

ANA-1 murine macrophage cells and TK6 human lymphoblastoid cells were maintained in Dulbecco's modified Eagle's media (Hyclone, Logan, UT) supplemented with 10% New Born Calf serum (NBCS) (Biofluids, Rockville, MD), 2 mM glutamine (Biofluids), penicillin (10 U/ml) and streptomycin (10 μg/ml, Biofluids) in growing suspension culture at 37°C in a humidified 5% CO_2_ atmosphere. Experiments with quinacrine were carried out by pre-incubating cells with indicated concentrations of quinacrine for specified times. Quinacrine was dissolved in DMEM medium (0.1% NBCS) containing 1% DMSO. Following a wash step, cells were activated by exposure to 100 U/ml interferon (IFN)-γ (R&D Systems, Minneapolis, MN).

### Western blot analysis and antibodies

Western blots were carried out as described previously [22, 57]. Antibodies used include: iNOS (Rabbit polyclonal, diluted 1 in 500, cat#160862; Cayman Chemicals, Ann Arbor, MI) and GAPDH (Rabbit monoclonal, diluted 1 in 1000, cat# 5174; Cell Signaling Technology, Danvers, MA). Horseradish peroxidase-conjugated anti-mouse and anti-rabbit secondary antibodies were purchased from Amersham Biosciences (Piscataway, NJ). Both secondary antibodies were diluted at 1:2000. All antibodies were diluted in 5% milk/PBST (0.1% Tween 20 in PBS). The Western blot signal was detected by Pierce ECL Western Blotting Substrate (Thermo Scientific, Rockford, IL) and developed onto Hyperfilm (GE Healthcare Life Sciences, Pittsburgh, PA). Briefly, after treating blot with the chemiluminescent substrate (Pierce ECL) for a minute, the blot was exposed to the hyperfilm in the dark (Exposure time was optimized based on the band signal obtained) and the film was developed in an automatic x-ray film processor (Futura Classic E automatic x-ray film processor, Fisher Industry, Geneva, IL).

### Flow-cytometric TUNEL analysis

TUNEL labeling was performed as we have done previously [25]. Briefly, TK6 cells were incubated in 0.1% NBCS supplemented RPMI-1640 media for 24 hrs. The media was changed and the cells were treated with vehicle (1% DMSO in PBS) or quinacrine (100 μg/ml). Cells were harvested after 12h of treatment and TUNEL assay was performed as described by vendor (Roche Diagnostics, IN).

### Animals

Male C57BL/6 mice, 8 weeks of age, weighing 20 to 25g were obtained from The Jackson Laboratories. All mice were kept in dedicated and clean animal quarters and provided food and water. Care and use of animals was overseen by the Animal Resource Facility (ARF) of the University of South Carolina under the direction of a veterinarian. The ARF is fully accredited by the Association for Assessment and Accreditation of Laboratory Animal Care International, is registered with the U.S. Department of Agriculture (56-R-003) and has an active letter of Assurance of Compliance on file at the NIH. Animal Care and Use Committee (IACUC) of the University of South Carolina approved this study.

The dextran sulfate sodium (DSS) mouse model used here is similar to the one used previously by our lab [24]. Animals received either water or 2% DSS dissolved in water for 7 days. Seven days after the initial DSS treatment, and after confirming quinacrine does not interact directly with DSS, we initiated a daily regimen of 50 mg/kg/ or 10 mg/kg of quinacrine dihydrocloride (Sigma) delivered in the drinking water containing 1% DMSO (doses were calculated assuming that the average adult mouse consumes 5 ml of water daily). DSS treatment continued in indicated groups. The doses of quinacrine were chosen based on the recommended dose in humans for treating systemic lupus erythematosis and giardiasis, which is 1.6 mg/kg (100 mg daily, with the assumption the average person weighs 60 kg). The animal equivalent to 1.6 mg/kg in humans is 20 mg/kg [58]. Other previous studies have also used similar doses in mice by gavage [59]. Following 7 or 10 days of treatment with quinacrine, the mice were sacrificed and the colon was harvested for analysis.

For our oxazolone model of UC we used a modified protocol generally following the methods described by Wirtz *et al.* [27]. Briefly, on day 0, the skin of the mice was treated with either 150 μl of oxazolone (Sigma, St Louis, MO) or 150 μl of vehicle control for pre-sensitization. The oxazolone presensitization solution is four parts acetone to one part olive oil containing 3% (wt/vol) oxazolone. The vehicle control was four parts acetone to one part olive oil alone. After 5 days, mice were weighed, anesthetized and either 100 μl oxazolone solution or 100 μl vehicle control was given by rectal administration. The oxazolone solution was 1% oxazolone mixed into a 50% ethanol solution. The vehicle control was 50% ethanol solution alone. Mice were held in a vertical head down position for 60 s and then put back into their cages. For long-term oxazolone treatment group of mice, we performed an additional 1% oxazolone rectal administration in 5 days. Quinacrine treatment was started in 8h after rectal oxazolone administration and continued daily. In 5 days after last 1% oxazolone administration, mice were euthanized and colons were processed for pathology and immunohistochemistry.

### Quantification of inflammation

The harvested colon was washed in PBS, Swiss-rolled, fixed in formalin and embedded in paraffin. The sections were then stained with hematoxylin and eosin. The slides were examined by two individuals in a blind fashion and the histopathologic changes were recorded using the previously described scoring system [24]. Inflammation was scored based on the extent and severity of inflammation and also crypt damage of the colonic tissue. Histology score was determined by multiplying the range of involvement (1 - 4) for each of these three histologic features by the percent area of involvement (0 - 100%) as described previously [24].

### Immunohistochemical staining

For immunohistochemical staining, serial sections of mouse colon tissues (processed as described above) were incubated with antibodies against p53 (Rabbit polyclonal, cat# 31333, diluted 1:1000; Abcam, Cambridge, MA), cyclooxygenase-2 (Cox-2) (Rabbit polyclonal, cat# 160126; diluted 1:5000; Cayman Chemical, Ann Arbor, MI) or inducible nitric oxide synthase (iNOS) (Rabbit Polyclonal, cat# 160862, diluted 1:3500; Cayman Chemical, Ann Arbor, MI). To ensure even staining and reproducible results, sections were incubated by slow rocking overnight in primary antibodies (4°C) using the Antibody Amplifier™ (ProHisto, LLC, Columbia, SC). Following incubation with primary antibody, sections were processed with EnVision+ System-HRP kits (DakoCytomation, Carpinteria, CA) according to the kit protocol. The chromogen was diaminobenzidene and sections were counter stained with 1% methyl green. The negative control was carried out without primary antibody incubation.

### Quantification of immunohistochemistry

Immunohistochemistry was quantified as we described previously [60], with a slight modification. The intensity of the staining was evaluated independently by two blinded investigators (A.C. and E.W.). For each tissue section, the percentage of positive cells was scored on a scale of 0 to 5 for the percentage of tissue stained: 0 (0% positive cells), 1 (<10%), 2 (11% to 25%), 3 (26% to 50%), 4 (51% to 80%), or 5 (> 80%). Staining intensity was scored on a scale of 0 to 3: 0 - negative staining, 1 - weak staining, 2 - moderate staining, or 3 - strong staining. The two scores were multiplied resulting in an immunoreactivity score (IRS) value ranging from 0 to 15.

### Quantification of clinical disease index (CDI)

CDI was assessed as described previously [61]. Briefly, mice were observed bi-daily for clinical signs of disease attributed by weight loss, fecal hemoccult and diarrhea during all treatments and till the final day of experiment. Ranking for the weight loss was based on the following scale: 0 = 0-5% weight loss; 1 = 6-10% weight loss; 2 = 11-15% weight loss; 3 = 16-20% weight loss; and 4 = >20% weight loss. The appearance of diarrhea was scored as: 0 = well-formed pellets, 2 = pasty and semi-formed stools that do not adhere to the anus, 4 = liquid stools that adhere to the anus. Appearance of blood in the stools was assessed using a hemoccult kit (Beckman Coutler) and scored as: 0 = no blood, 2 = positive hemoccult, 4 = gross bleeding. The clinical score was then determined by totaling the weight loss, hemoccult, and diarrhea scores with the highest score being twelve.

### Statistical analysis

With inflammation as an end point, a χ2 contingency table analysis was done on the DSS and DSS + Quinacrine groups to determine if there was a statistically significant difference in their inflammation scores.
